# Rice Seeds as Biofactories of Rationally Designed and Cell-Penetrating Antifungal PAF Peptides

**DOI:** 10.3389/fpls.2019.00731

**Published:** 2019-06-07

**Authors:** Mireia Bundó, Xiaoqing Shi, Mar Vernet, Jose F. Marcos, Belén López-García, María Coca

**Affiliations:** ^1^Centre for Research in Agricultural Genomics (CRAG, CSIC-IRTA-UAB-UB), Barcelona, Spain; ^2^Institute of Agrochemistry and Food Technology (IATA, CSIC), Paterna, Spain

**Keywords:** PAF, antifungal, pathogens, fungi, rice, seed, oil bodies, protein bodies

## Abstract

PAFs are short cationic and tryptophan-rich synthetic peptides with cell-penetrating antifungal activity. They show potent and selective killing activity against major fungal pathogens and low toxicity to other eukaryotic and bacterial cells. These properties make them a promising alternative to fulfill the need of novel antifungals with potential applications in crop protection, food preservation, and medical therapies. However, the difficulties of cost-effective manufacturing of PAFs by chemical synthesis or biotechnological production in microorganisms have hampered their development for practical use. This work explores the feasibility of using rice seeds as an economical and safe production system of PAFs. The rationally designed PAF102 peptide with improved antifungal properties was selected for assessing PAF biotechnological production. Two different strategies are evaluated: (1) the production as a single peptide targeted to protein bodies and (2) the production as an oleosin fusion protein targeted to oil bodies. Both strategies are designed to offer stability to the PAF peptide in the host plant and to facilitate its downstream purification. Our results demonstrate that PAF does not accumulate to detectable levels in rice seeds when produced as a single peptide, whereas it is successfully produced as fusion protein to the Oleosin18, up to 20 μg of peptide per gram of grain. We show that the expression of the chimeric *Ole18-PAF102* gene driven by the *Ole18* promoter results in the specific accumulation of the fusion protein in the embryo and aleurone layer of the rice seed. Ole18-PAF102 accumulation has no deleterious effects on seed yield, germination capacity, or seedling growth. We also show that the Oleosin18 protein serves as carrier to target the fusion protein to oil bodies facilitating PAF102 recovery. Importantly, the recovered PAF102 is active against the fungal phytopathogen *Fusarium proliferatum*. Altogether, our results prove that the oleosin fusion technology allows the production of PAF bioactive peptides to assist the exploitation of these antifungal compounds.

## Introduction

Infections caused by fungi pose a serious threat to human and animal health and to food security and safety (Fisher et al., 2012). Invasive fungal diseases have significantly increased in recent decades and are important causes of mortality, particularly in immunocompromised patients, killing about one and a half million people every year. This value exceeds the death rate for malaria or breast cancer (Brown et al., 2012). Plant disease epidemics caused by fungi and fungal-like oomycetes are an old problem that have been further exacerbated by intensive agricultural practices, globalization, and climate change (Bebber and Gurr, 2015). Today, crop-destroying fungi account globally for yield losses of ~20%, with further 10% postharvest losses (Fisher et al., 2018). In addition, food safety is challenged by mycotoxigenic fungi that contaminate food and feed with detrimental toxins for human health. The limited number of licensed antifungals currently available, together with the unprecedented rise of multidrug-resistant pathogenic fungi, makes crucial the development of novel antifungal compounds to combat fungal infections (Perfect, 2016; Fisher et al., 2018). Antimicrobial peptides (AMPs) are being actively explored to fulfill the need of novel antifungals with potential applications in crop protection, food preservation, and medical therapies.

AMPs are peptides and small proteins produced by most living organisms that exhibit lytic or inhibitory activity against microorganisms (Zasloff, 2002; Zhang and Gallo, 2016). However, most AMPs are obtained at low yields from natural sources, and some of them show properties, such as low stability or low specificity, that might compromise their applications. Their peptidic nature enables the rational design of novel molecules with improved properties to be produced at high yields through biotechnological systems. PAF102 was designed as a novel antifungal peptide with improved properties and optimized to be produced in biofactories (López-García et al., 2015). PAF102 is a modified concatemer of the hexapeptide PAF26 (RKKWFW), which was identified through a combinatorial approach as a Peptide with specific AntiFungal activity (PAF) (López-García et al., 2002, 2015; Muñoz et al., 2013). PAF102 shows potent antifungal activity against economically relevant phytopathogens and very low toxicity to other eukaryotic cells, including human erythrocytes. The antifungal mechanism of PAF102 is similar to that of the parental PAF26, and it involves the interaction with the fungal cell envelope, followed by cell penetration and intracellular effects that cause cell death (Muñoz et al., 2013; López-García et al., 2015). This mode-of-action is different to the one of licensed antifungal drugs; thus, PAF peptides might be an alternative to combat fungal pathogens. However, the difficulties of cost-effective manufacturing of PAFs by chemical synthesis, or by conventional microbial-based production systems associated to host toxicity, have hampered their development for practical use.

Plants provide a platform for the production of AMPs that offer advantages in terms of cost-effectiveness and scalability as they are economical and easy to grow, as well as of safety because of the low risk of contamination with human and animal pathogens (Twyman et al., 2003). Particularly, rice seeds have been reported as efficient bioreactors of AMPs, including natural or rational designed peptides (Bundó et al., 2014; Montesinos et al., 2016, 2017). AMP production is favored by limiting their accumulation to seeds that avoids the negative impacts on plant performance reported in some cases when accumulated in vegetative tissues (Coca et al., 2006; Nadal et al., 2012; Company et al., 2014). Several seed-specific promoters are now available to drive strong expression of *AMP* genes either in the rice endosperm or embryo (Qu and Takaiwa, 2004). Among endosperm-specific promoters are the ones from the seed storage proteins glutelins and globulins, including the *GluB1*, *GluB4*, and *Glb1* promoters; and among embryo-specific promoters are the ones from the oleosin proteins, such as the *Ole18* promoter. Another factor that favors the production of AMPs in seeds is their confinement into storage organelles, such as protein bodies (PBs) or oil bodies (OBs) (Bundó et al., 2014; Montesinos et al., 2016, 2017). Storage organelles offer a stable environment for packing a large amount of AMPs, together with host cell protection from AMP exposure. Proteins can be targeted to PBs through signal peptides linked at their N-terminus and/or KDEL sequence at their C-terminus, together through intrinsic physicochemical properties in certain storage proteins (Khan et al., 2012; Takaiwa et al., 2017). OB targeting is achieved using oleosin proteins as carriers (van Rooijen and Moloney, 1995; Montesinos et al., 2016). Oleosins are the most abundant structural proteins of plant seed OBs, whose lipophilic character and secondary structure determine their association to OBs (Abell et al., 1997). Both PBs and OBs have served to stabilize AMPs in rice seeds and to reach high yields (Bundó et al., 2014; Montesinos et al., 2016).

This work explores the feasibility of using rice seeds as a platform for the production of cell-penetrating antifungal PAF peptides, exemplified as PAF102. Two different strategies are evaluated: (1) the production as a single peptide targeted to PBs or (2) the production as an oleosin fusion protein targeted to OBs. Here, we report that PAF103, a His-tagged and KDEL-extended PAF102, was not accumulated to detectable levels in rice seeds, whereas PAF102 was successfully produced as fusion to the Ole18 protein in rice seeds. We demonstrate that the Ole18-PAF102 fusion protein was accumulated in OBs without affecting seed yield or germination capacity. We also show that biologically active PAF102 can be recovered from rice OBs. Our results demonstrate that the oleosin fusion technology is a good strategy for the production of PAF antifungal peptides.

## Materials and Methods

### Preparation of Plant Expression Vectors

**Figure 1:**
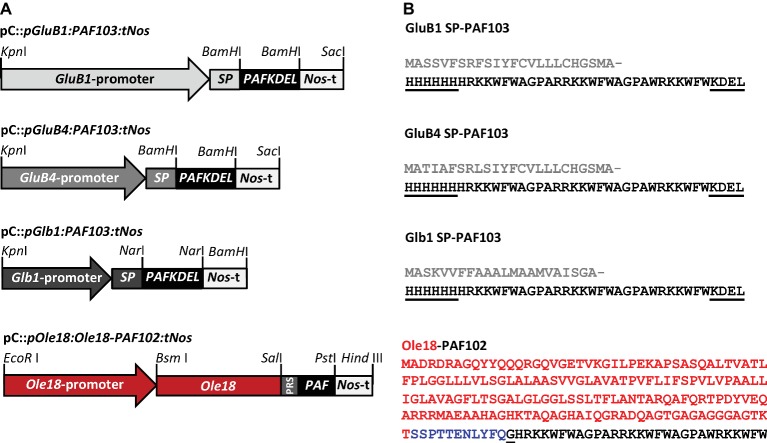
Gene constructs for PAF production in rice seeds. **(A)** Schematic representation of the constructs in which the expression of the synthetic *PAF103* gene was controlled by the 2.3 kb *GluB1*, or the 1.4 kb *GluB4,* or 0.9 kb *Glb1* promoters, and the expression of the chimeric *Ole18-PAF102* fusion gene by the *Ole18* promoter. The corresponding signal peptides (SP) were included in the PAF103 constructs. The *Nos* terminator (Nos-t) was present in all the constructs. PRS corresponds to the TEV protease recognition sequence that links the Ole18 protein to PAF102. Relevant restriction enzyme sites for cloning purposes are indicated. **(B)** Amino acid sequences of PAF103 peptide and Ole18-PAF102 fusion protein. Gray sequences correspond to SPs of the corresponding storage proteins, which are post-translationally cleaved; black sequences to the mature PAF103 or PAF102 peptides; red sequence to the 18 kDa oleosin (Ole18) protein; and blue sequence to PRS. Underlined sequences correspond to the differential residues between PAF peptides.

Four different constructs were prepared for the expression of the synthetic *PAF* genes in rice seeds (Figure 1A). Three of them were designed for the production of a PAF as an individual peptide, and the last one as a fusion to the rice Oleosin 18 kDa protein (Ole18). The individual peptide was His-tagged in N-terminal and KDEL-extended in C-terminal resulting in a new PAF that was named PAF103 (Figure 1B). The *PAF103* gene was synthesized by GenScript based on the codon usage bias in *Oryza sativa* and flanked in both ends by *BamH*I restriction sites (Supplementary Figure S1). To drive the expression of *PAF103*, three different endosperm-specific promoters were used, namely *Glutelin B1* (*GluB1*), *Glutelin B4* (*GluB4*), and the 26 KDa *Globulin* (*Glb1*) (Qu and Takaiwa, 2004). Additionally, the sequence encoding the signal peptide of the corresponding seed storage proteins was fused to the N-terminus of the *PAF103* gene for internalization into the endoplasmic reticulum (ER) system (Figure 1B). The vectors containing the *pGluB1:PAF103:tNos* and *pGluB4:PAF103:tNos* constructs were prepared replacing the *BamH*I-*BamH*I *CecA-KDEL* DNA fragment by the *BamH*I-*BamH*I *PAF103* DNA fragment into the previously described pC::*pGluB1:CecAKDEL:tNos* and pC::*pGluB4:CecAKDEL:tNos* vectors (Bundó et al., 2014). The pC::*pGlb1:PAF103:tNos* vector was prepared by replacing the *Nar*I-*Nar*I *BP178* fragment by a *Nar*I-*Nar*I *PAF103* fragment into the previously described pC::*pGlb1:BP178:tNos* vector (Montesinos et al., 2017). The *Nar*I-*Nar*I *PAF103* fragment was obtained by PCR amplification from the GenScript clone using the oligonucleotides NarIPAF103_fwd and PAF103NarI_rev in Supplementary Table S1.

An additional vector for the expression of the chimeric gene encoding an Ole18-PAF102 fusion protein was prepared (Figure 1A). In this case, the gene expression was driven by the embryo-specific *Ole18* own promoter (Montesinos et al., 2016). The fusion protein corresponds to the Ole18 protein linked to the PAF102 peptide through a Tobacco Etch Virus NIa protease recognition site (TEV protease) (Figure 1B), without His-tag or KDEL extension but with an extra glycine residue at N-terminus that remains after TEV protease digestion. The construct was prepared by PCR amplification of the *Ole18* promoter and the Ole18 protein coding sequence (*pOle18:Ole18*) from the pC::*pOle18:Ole18-CecA:tNos* vector (Montesinos et al., 2016) using the primers in Supplementary Table S1, which introduce an *EcoR*I and a *Sal*I site at 5′ and 3′ ends of the fragment, respectively. The PAF102 coding sequence extended in frame at N-terminus with the TEV protease recognition site (PRS), and flanked by *Sal*I and *Pst*I restriction sites, was synthesized by GenScript (Supplementary Figure S1). The *Nopaline Synthase* (Nos)-terminator sequence was introduced into the cloning vector containing the *PRS-PAF102* (pUC57::*PRS-PAF102*) as a *Pst*I-*Hind*III fragment, which was amplified by PCR using the primers that add these restriction sites (Supplementary Table S1). Into this plasmid, the *pOle18:Ole18* fragment was also introduced as an *EcoR*I-*Sal*I restriction fragment. Finally, the whole construct was mobilized to the binary vector pCAMBIA1300 as an *EcoR*I-*Hind*III fragment to generate the pC::*pOle18:Ole18-PAF102:tNos* vector for rice transformation (Figure 1A). All the constructs were then verified by nucleotide sequencing.

### Production of Transgenic Plants

Transgenic rice plants (*Oryza sativa* cv. Ariete) were produced by *Agrobacterium*-mediated transformation of embryogenic calli as previously described (Sallaud et al., 2003). Transgene insertion was confirmed in the regenerated plants by PCR analysis using leaf genomic DNA as template. The positive plants were selected to obtain homozygous lines in the T2 generation. The homozygous lines were identified by segregation of hygromycin resistance afforded by the *htp*II marker gene in the T-DNA region of pCAMBIA1300-derived vectors. The transgene copy number was estimated by quantitative PCR (qPCR) using the *Sucrose Phosphate Synthase* (*SPS*) reference gene as previously described (Yang et al., 2005; Bundó et al., 2014). Rice plants transformed with the empty vector (pCAMBIA 1300) were also produced as a control for this study. All rice plants were grown at 28 ± 2°C with a 14/10 h light/dark photoperiod.

### Protein Extraction and Immunoblot Analysis

Protein extracts were prepared from dehulled mature seeds (10 seeds, ~200 mg) imbibed in water for 1 h. Seeds were ground and homogenized in a sucrose-containing buffer (10 mM phosphate buffer pH 7.6, 0.6 M sucrose). After filtration with miracloth, homogenates were centrifuged at low speed (200*g*) to remove cellular debris and starch. Clarified homogenates were then centrifuged at high speed (2,000*g*) to obtain PB-enriched fractions, as the precipitated dense fractions (Bundó et al., 2014), or the OB-enriched fractions, as the floating fractions (Montesinos et al., 2016). PB-enriched fractions were resuspended directly in SDS-loading buffer, separated on tricine-SDS-PAGE (16.5%), transferred to a nitrocellulose membrane (Amersham Protran 0.2 μm), and immunodetected using commercial monoclonal antibodies anti-His tag (A00186 GeneScript).

Immunoblot analysis of OB-associated proteins was done after solubilization in SDS-loading buffer, separation in SDS-PAGE, transfer to nitrocellulose membranes (Amersham Protran 0.4 μm), and immunodetection with antibodies against the PAF102 (1:1,000 dilution, this work) and the rice Ole18 [1:2,000 dilution, (Montesinos et al., 2016)]. Mouse polyclonal antibodies against synthetic PAF102 (GeneScript) were produced at the Laboratory Animal Facilities (registration number B9900083) of the Center for Research and Development (CID) from the Spanish National Research Council (CSIC), in strict accordance with the bioethical principles established by the Spanish legislation following international guidelines. The protocol was approved by the Committee on Bioethics of Animal Experimentation from CID and by the Department of Agriculture, Livestock, Fisheries, Food and Environment of the Government of Catalonia (permit number DAAM:7461). All efforts were made to minimize suffering of the animals. Four injections of synthetic PAF102 (0.5 mg each) in a three-weekly basis were applied to mice, which were bleed 1 week after the last injection to obtain the PAF102 antiserum.

The amount of PAF102 accumulation per seed was estimated on immunoblot by quantification of signal intensities of Ole18-PAF102 to known amounts of synthetic PAF102. Signal intensities were quantified using the Quantity Tools Image Lab™ Software (Version 5.2.1) included in the ChemiDoc™ Touch Imaging System (Bio-Rad, USA).

### RT-PCR Analysis of Transgene Expression

Transgene expression was determined by RT-PCR analysis of total RNA isolated from a pool of 10 immature seeds (before seed desiccation, around 20–25 days after flowering). Total RNA was extracted using the method previously described (Chang et al., 1993). DNase-treated RNA (1 μg) was retrotranscribed using the High Capacity cDNA Reverse Transcription kit (Applied Biosystems) using the oligo(dT) primer. PCRs were carried out using specific primers (Supplementary Table S1) that annealed to GluB1, GluB4, and Glb1 SP encoding sequences (forward primers) and to the *PAF103* gene sequence (reverse primers). Amplified transcripts encoded by the three different transgenes were compared to the *OsEF1a* housekeeping transcripts (Os03g08060).

### *In situ* Immunodetection of PAFs in Whole Seeds

PAF103 and PAF102 accumulation in the transgenic rice seeds was analyzed by *in situ* immunodetection using the anti-His tag (dil 1:1,000) and anti-PAF102 antibodies (dil 1:500), respectively, and the fluorescent labeled AlexaFluor448 anti-mouse secondary antibody (Molecular Probes, 1:5,000 dilution) as previously described (Bundó et al., 2014).

### Fungal Infection Assays on Rice Seeds

Transgenic rice seeds were evaluated for resistance to the seed fungal pathogen *Fusarium proliferatum* as previously described (Bundó et al., 2014). Briefly, 12 surface-sterilized seeds per line and treatment were placed on MS medium without sucrose and then inoculated with 50 μl of sterile water (control) or of *F. proliferatum* spore suspension (10^3^ spores/ml). Seeds were allowed to germinate for 7 days to determine the percentage of germination under control or infection conditions. Three independent assays were performed.

### OBs Isolation and PAF102 Purification

OBs were isolated from the OB-enriched fractions by two consecutive cycles of flotation-centrifugation on a sucrose containing buffer. The integrity of the isolated OBs was tested by selective staining with Nile red (1 ng/ml, Sigma) and confocal fluorescent visualization. PAF102 was recovered from the OBs containing the Ole18-PAF102 protein by digestion with TEV protease (Invitrogen, 1:100). Proteolytic digestion was conducted overnight at 30°C in the TEV protease buffer supplemented with 0.25 M sucrose. TEV protease efficiency was estimated based on the disappearance of the Ole18-PAF102 signal in immunoblot analysis by quantification of signal intensities.

### Antifungal Assays

Growth inhibition assays of *F. proliferatum* were performed in 96-well flat-bottom microtiter plates, as previously described (López-García et al., 2015). Basically, 70 μl of fungal conidia (1.4 × 10^3^ conidia/ml) in half strength of potato dextrose broth (PDB) containing 0.02% chloramphenicol were mixed in each well with 30 μl of samples in OB resuspension buffer (0.2 M sucrose; 10 mM Tris-HCl pH 7.5; 0.02% Tween-20). Samples were prepared in triplicate. Plates were incubated with agitation for 72 h at 28°C. Fungal growth was monitored every 24 h by measuring the optical density (OD) at 600 nm using a Spectramax M3 reader (Molecular Devices), and mean values and standard deviation (SD) were calculated. Experiments were repeated twice.

## Results

### Generation and Characterization of Transgenic Rice Plants

Three different constructs were prepared for the expression of a *PAF* synthetic gene in rice seed endosperm (Figure 1A). Two of them contain the glutelin promoters (*pGluB1* and *pGluB4*) to drive expression in the peripheral region of the endosperm, and the other one contains the 26 kDa globulin promoter (*pGlb1*) to drive expression in the inner starchy endosperm tissue (Qu and Takaiwa, 2004). All three constructs were designed to produce an individual PAF peptide targeted to PBs by including the signal peptides (SP) of the corresponding storage protein and the KDEL signal. The N-terminal signal peptides target proteins to the secretory pathway, are co-translationally cleaved, and are indispensable for PB sorting (Takagi et al., 2005b). However, the C-terminal endoplasmic reticulum (ER) retention sequence (KDEL) remains in the mature proteins, and although it is not strictly required for PB deposition, it is reported to favor accumulation levels (Takagi et al., 2005b). In addition to the KDEL sequence, the PAF102 was His-tagged at the N-terminus to facilitate its purification from rice endosperm, resulting in a new PAF peptide that was named PAF103 (Figure 1B). Growth inhibitory activity against the *F. proliferatum* fungal pathogen revealed equivalent antifungal activity for both peptides, namely PAF102 and PAF103, with a minimal inhibitory concentration (MIC) value of 3.5 μM.

One additional construct was prepared to produce PAF102 as a fusion protein to the Ole18 (Figure 1A). In this case, the expression of the chimeric fusion gene *Ole18-PAF102* is directed by the embryo specific promoter of the *Ole18* gene (Qu and Takaiwa, 2004). This strategy intends to target PAF102 to OBs.

**Figure 2:**
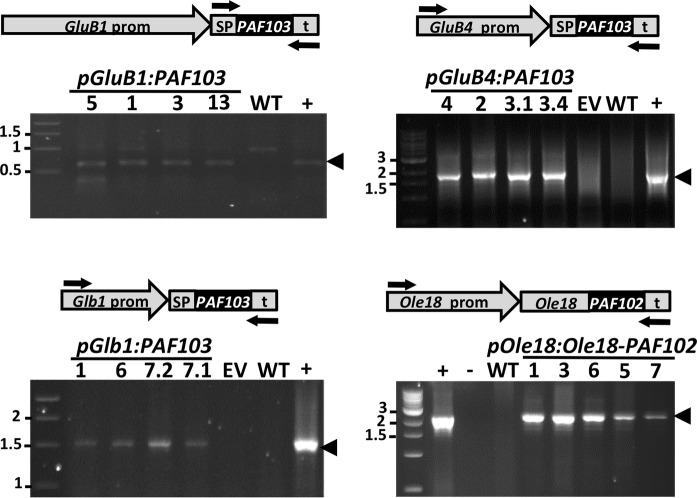
Detection of the transgenes in the rice plant genome. PCR analysis on genomic DNA purified from leaves of wild-type (WT) or transgenic lines carrying the empty vector (EV) or the indicated transgenes. Plasmid DNA was used as a positive control (+). Arrows indicate the position of the specific oligonucleotides used for PCR amplification. The size of amplified fragments showed full length transgene insertion. Molecular markers are shown on the left in kpb.

Transformation of embryogenic rice calli was performed *via Agrobacterium tumefaciens*. Using the hygromycin resistance for selecting transformed calli, a similar number of plants was regenerated for each transformation event (around 10 independent lines obtained from independent calli). The presence of the transgenes was confirmed in most of the regenerated plants by PCR analysis using leaf genomic DNA as template. The positive plants were grown under containment greenhouse conditions to obtain homozygous transgenic lines in T2 generation. Four to five independent homozygous lines per construct were identified based on the segregation of the hygromycin resistance marker. No apparent adverse effects on growth, flowering, or grain yield were observed on the transgenic PAF plants across generations; all of them performed similar to the plants transformed with the empty vector grown simultaneously under the same conditions. Stability and inheritance of transgenes in T3 plants were then confirmed by PCR analysis of genomic DNA, as done in the T0 plants (Figure 2). The copy number of transgene insertions in the different lines was estimated by qPCR analysis in comparison to the *SPS* single copy gene in the rice genome. The results show that the transgenes were present in a single copy in all the selected homozygous lines, in agreement with the segregation in previous generations of the antibiotic resistance marker encoded in the T-DNA (Supplementary Table S2).

### PAF103 Does Not Accumulate in PBs of Rice Seeds

The accumulation of the single PAF103 peptide was evaluated on T2 homozygous seeds carrying the constructs *pGluB1:PAF103*, *pGluB4:PAF103*, or *pGlb1:PAF103*. Seed protein extracts enriched in dense organelles were successfully used previously for detection of cationic antimicrobial peptides deposited in PBs, such as cecropin A or BP178 (Bundó et al., 2014; Montesinos et al., 2017). Accordingly, we prepared PB-enriched extracts from mature seeds of all the homozygous lines carrying the constructs for *PAF103* expression. Western blot analysis using anti-His tag antibodies for PAF103 detection revealed no differential band on protein extracts from transgenic lines in comparison to wild type, whereas PAF103 was clearly immunodetected on a wild-type extract supplemented with the synthetic PAF103 peptide (Supplementary Figure S2). This result suggests that PAF103 does not accumulate in the transgenic seeds. To discard an extraction problem, we conducted an *in situ* immunodetection assay in whole seeds that equally failed to detect PAF103 on the transgenic seeds.

A different approach to assess PAF103 accumulation is through the detection of its antifungal activity. Thus, transgenic *PAF103* seeds were then evaluated for resistance to *F. proliferatum*. However, *PAF103* seeds showed similar, or even increased, susceptibility to the fungal pathogen than wild-type or empty vector seeds (Supplementary Figure S3). All the seeds from the different lines showed reduced germination capacity and reduced seedling growth after inoculation with fungal spores, whereas they germinated and grew normally under control conditions. Therefore, the antifungal activity of PAF103 was not detected in the transgenic rice seeds, providing additional support that the PAF103 peptide is not accumulated in these seeds.

**Figure 3:**
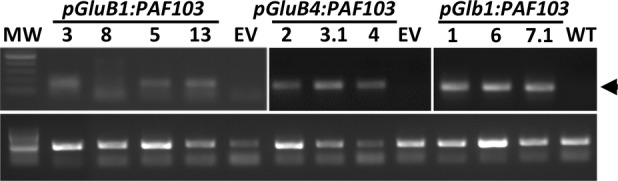
*PAF103* transgene expression in rice seeds. RT-PCR analysis of indicated transgenes in immature rice seeds. PAF103 transcripts (upper bands) were compared to the OsEF1a housekeeping gene transcripts (lower bands).

Given that transgenes were integrated in the genome of all the independent transgenic *PAF103* lines but the transgene products were not detected, we evaluated the transgene expression in immature seeds at the developmental stage where *GluB1*, *GluB4*, and *Glb1* promoters are active (Qu and Takaiwa, 2004). We amplified the corresponding mRNAs in the tested independent lines (Figure 3) by RT-PCR analysis on total RNA isolated from seeds and using specific primers (Supplementary Table S1). These results indicate that although *pGluB1:PAF103*, *pGluB4:PAF103*, or *pGlb1:PAF103* transgene is expressed in the rice seeds, the corresponding product PAF103 does not accumulate to detectable levels.

### PAF102 Accumulates as Fusion to the Ole18 Protein in Rice Seeds

**Figure 4:**
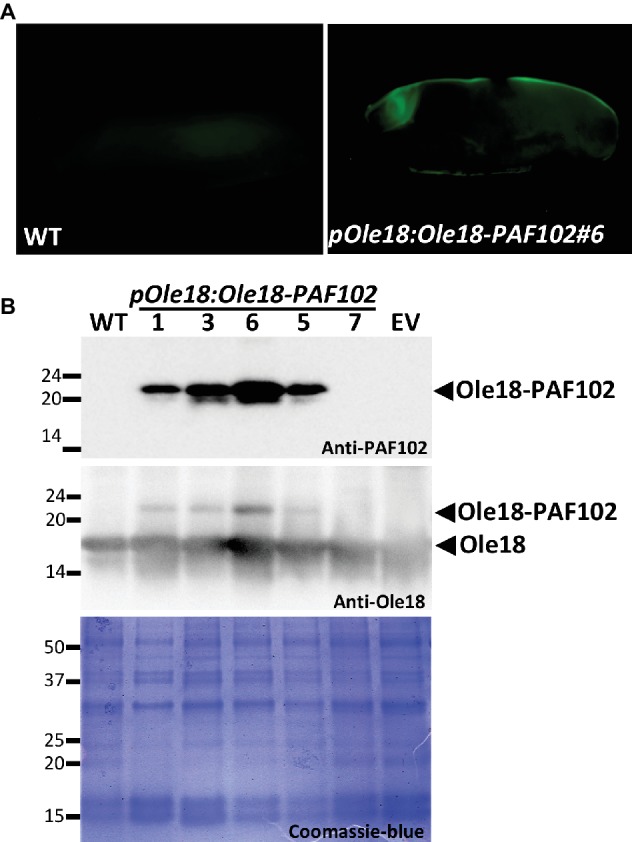
PAF102 accumulates stably in rice seeds as an oleosin fusion protein. **(A)** *In situ* immunolocalization of PAF102 in *pOle:Ole18-PAF102* transgenic seeds (line #6) in comparison to wild-type (WT). Immunoreaction was detected using a fluorescent-labeled secondary antibody. **(B)** Immunoblot analysis of OB fractions purified from seeds of WT, empty vector (EV), and five independent transgenic lines carrying the *pOle18:Ole18-PAF102* transgene, using anti-PAF102 or anti-oleosin18 antibodies as indicated. Protein profile of OB fractions is shown by Coomassie blue staining of SDS gel. Proteins were purified from recent harvested seeds.

The accumulation of the PAF102 when fused to the Ole18 protein was evaluated in the T2 homozygous seeds of transgenic lines carrying the *pOle18:Ole18-PAF102*. We first analyzed PAF102 accumulation by *in situ* immunodetection in whole mount seeds. As shown in Figure 4A, PAF102 was immunodetected in the seed embryo and aleurone layer of *pOle18:Ole18-PAF102* seeds but not in wild-type seeds. This distribution pattern corresponds to the expression pattern of the *Ole18* promoter (Qu and Takaiwa, 2004) and correlates with OB accumulation in rice seeds (Montesinos et al., 2016).

In order to confirm that PAF102 was produced as a fusion protein and retains the natural targeting of Ole18, we isolated OBs from seeds of five independent *pOle18:Ole18-PAF102* homozygous lines. After solubilization, OB-associated proteins were separated by SDS-PAGE and immunodetected using anti-PAF102 antibodies. A polypeptide of apparent molecular mass of 23 kDa (the expected mass of the fusion protein is 23.14 kDa, corresponding to 18 kDa of oleosin + 1.2 kDa of TEV protease recognition size + 3.94 kDa of PAF102) was clearly detected in the OBs of four out of five *pOle18:Ole18-PAF102* lines and was absent in the empty vector and wild-type OBs (Figure 4B). This protein was also immunoreacting with the anti-Ole18 antibodies as an additional and less intense band than the one corresponding to the Ole18 protein. The accumulation of the fusion protein seems not to alter the protein profile of OBs as visualized by protein Coomassie blue staining. These results demonstrate that PAF102 accumulates in rice OBs when fused to the Ole18 protein.

The amount of produced PAF102 was estimated in T3 seeds in comparison to known amounts of PAF102. The highest value was found in the line #6 producing 20 ± 3 μg/g of seed, and the mean value for the four independent lines was 15 ± 6 μg/g of seed.

In addition to be stably produced across plant generations, the fusion protein remains stable in seeds during long-time storage at room temperature. The fusion protein was still detected in seeds after 3 years of storage in the laboratory with a mean value of 16 ± 6 μg/g of seed.

### Ole18-PAF102 Accumulation Has No Negative Impact in Rice Plant Performance

**Figure 5:**
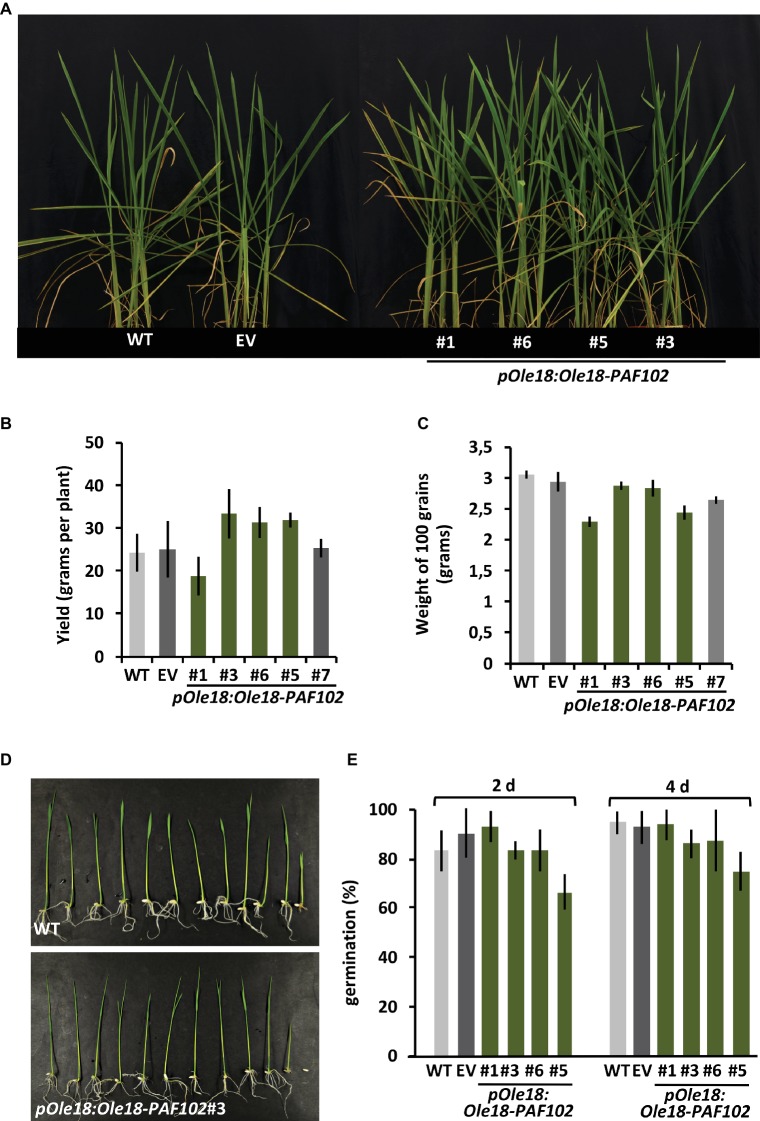
Production of the Ole18-PAF102 fusion protein in seeds has no negative impact in rice plant performance. **(A)** Phenotypical appearance of the wild-type (WT) and the transgenic rice plants carrying the empty vector (EV) or the *pOle18:Ole18-PAF102* gene at 30 days after sowing. **(B)** Average grain yield per plant calculated from four plants per line in three independent assays (*n* = 12). **(C)** Average weight of 100 seeds per line (*n* = 12). **(D)** Phenotypical appearance of seedlings at 7 days post imbibition. **(E)** Percentage of germinated seeds at 2 and 4 days after imbibition. Values correspond to the mean value of three independent assays. Error bars represent standard deviation.

PAF102 is a cell-penetrating peptide that kills fungal cells intracellularly (López-García et al., 2015). In order to evaluate potential toxicity of PAF102 fused to Ole18 and accumulated inside the embryonic rice cells, we characterized phenotypically the transgenic rice plants expressing the *Ole18-PAF102* under the control of the *Ole18* promoter. These plants showed normal phenotypical appearance during the vegetative phase, similar to the wild-type and empty vector plants (Figure 5A). Interestingly, they did not show a penalty in grain yield (Figure 5B), indicating that the accumulation of the Ole18-PAF102 does not affect seed production. Although differences in seed weight were observed among lines, those seeds accumulating the highest levels of Ole18-PAF102 showed similar weight on average to the control wild-type and empty vector seeds (Figure 5C). These data indicate that accumulation of the Ole18-PAF102 has no impact in seed filling. Additionally, seeds accumulating the recombinant fusion protein germinated at the same rate and timing as the control seeds, and their seedlings showed similar appearance (Figures 5D,E). Thus, the presence of Ole18-PAF102 in the OBs seems not to affect the viability of rice seeds and seedling growth. Altogether, these results suggest that the expression of *pOle18:Ole18-PAF102* does not alter the fitness of the rice plants.

### Ole18-PAF102 Accumulation Does Not Protect Rice Seeds Against Fungal Infection

**Figure 6:**
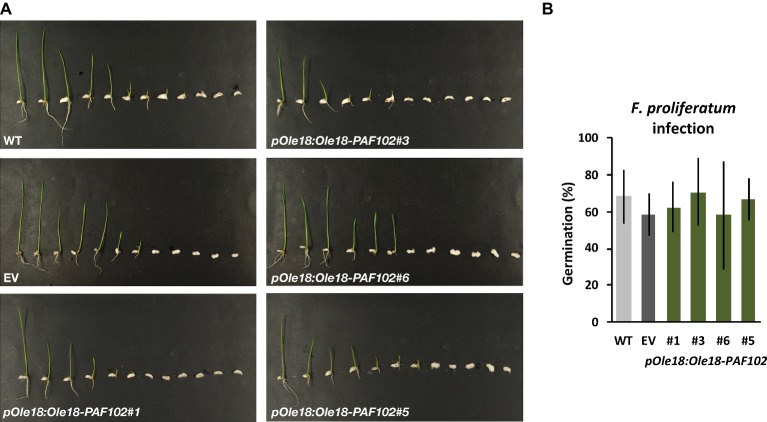
Ole18-PAF102 accumulation does not protect rice seeds against the fungal pathogen *F. proliferatum.* **(A)** Phenotypical appearance of wild-type (WT), empty vector (EV), and *pOle18:Ole18-PAF102* transgenic seedlings (lines #1, #3, #6, and #5) at 7 days after inoculation with *F. proliferatum* spore suspensions (10^3^ spores/ml). Pictures are representative of three independent experiments. **(B)** Percentage of seed germination upon infection in comparison to control conditions (see Figure 5D). The graph shows mean and standard deviation values of the indicated lines from three independent assays.

To investigate whether the fusion protein Ole18-PAF102 retains the antifungal activity of the single PAF102 peptide, we evaluated the *pOle18:Ole18-PAF102* seeds for resistance to *F. proliferatum*. These transgenic seeds showed similar susceptibility to the fungal pathogen than the wild-type or empty vector seeds (Figure 6). All the seeds from the different lines showed reduced germination capacity and reduced seedling growth after inoculation with fungal spores. These data suggest that the Ole18-PAF102 does not protect plants *in situ* against the fungal infection.

### Biologically Active PAF102 Is Recovered From pOle18:Ole18-PAF102 Seeds

**Figure 7:**
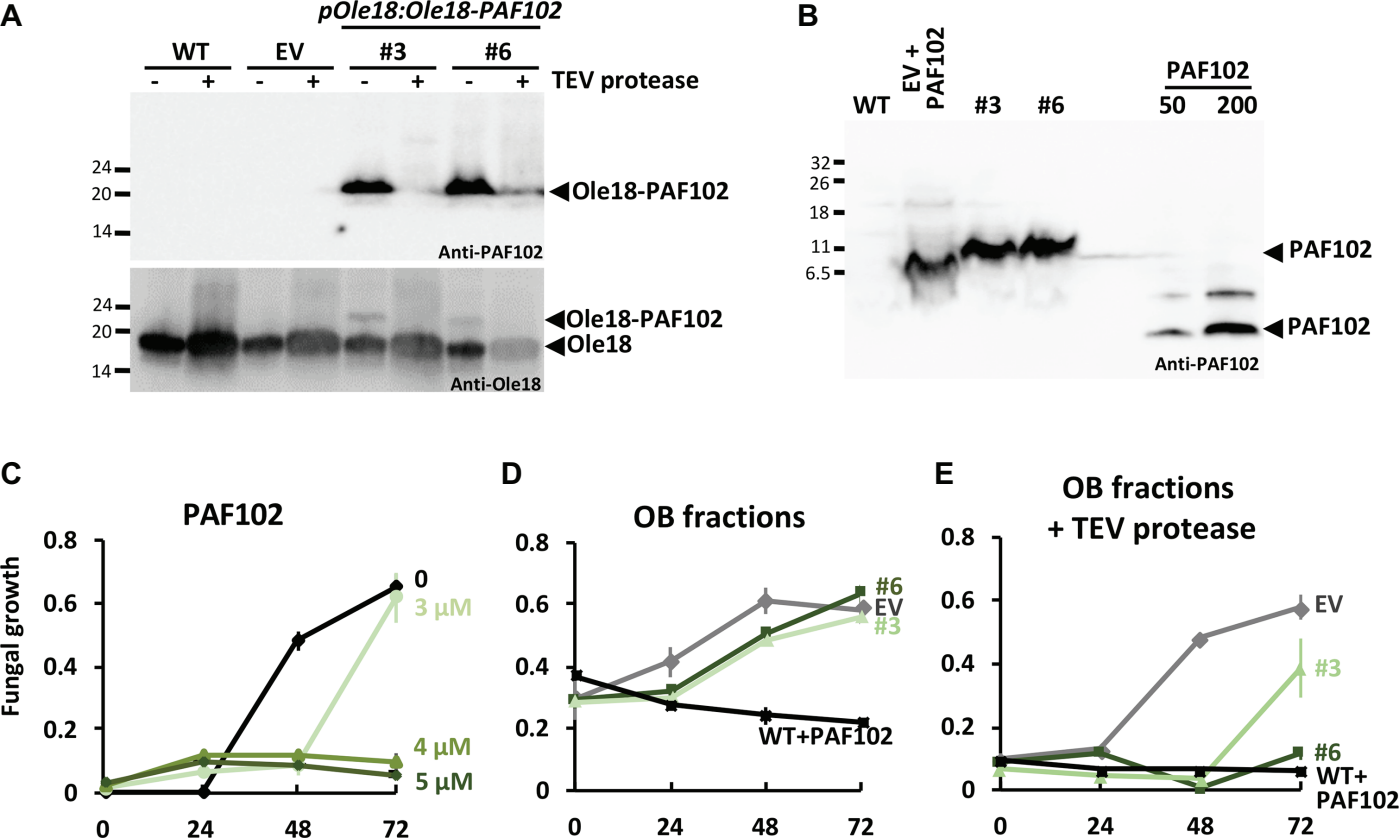
Recovery of active PAF102 from rice seed OBs. **(A,B)** Immunoblot analysis of equivalent amount of OB fractions from wild-type (WT), empty vector (EV), and *pOle18:Ole18-PAF102* lines #3 and #6 seeds, before (−) or after (+) TEV protease digestion, and using anti-PAF102 or anti-Ole18 antibodies. Digested fractions were run in a tricine-SDS gel in parallel to synthetic PAF102 (50 and 200 ng) or to synthetic PAF102 (200 ng) added to EV fractions **(B)**. **(C–E)** *In vitro* growth inhibitory activity of *F. proliferatum* by synthetic PAF102 peptide at indicated concentrations **(C)**, by OB fractions **(D)**, or by OB fractions digested with TEV protease **(E)** from indicated lines. WT fractions were supplemented with 5 μM of synthetic PAF102. Graphs show the mean of OD_600nm_ of triplicate samples ± SD.

We next assessed the recovery of the single PAF102 peptide from rice seed OBs carrying Ole18-PAF102. For that, we digested the recombinant OBs with the TEV protease, since Ole18 and PAF102 polypeptides were linked through the protease recognition site. The immunoblot analysis of OB fractions before and after proteolytic digestion is shown in Figure 7A. We observed that the fusion protein nearly disappeared after protease digestion of the OBs from two *pOle18:Ole18-PAF102* transgenic lines (#3, #6). Subtle differences were detected among lines and experiments, and TEV protease efficiency was calculated at 87.5 ± 6.5% on average. These data indicate a high efficiency of proteolytic processing of the fusion protein Ole18-PAF102 on intact OBs. Next, we investigated the presence of the PAF102 single peptide in the protease-digested fractions. We immunodetected a polypeptide in the fractions of lines #3 and #6 with a higher electrophoretic mobility to the synthetic PAF102 peptide, but that was absent in the WT fractions (Figure 7B). Equally, the polypeptide immunodetected in the EV fractions supplemented with synthetic PAF102 peptide showed a different mobility than the synthetic PAF102 peptide alone. These results indicate that, in the presence of plant extracts enriched with OBs, PAF102 exhibits different electrophoretic mobility, consistent with the altered electrophoretic mobility that has been previously reported for other small cationic peptides (Coca et al., 2006; Bundó et al., 2014; Montesinos et al., 2016, 2017). We also observed a couple of immunoreactive bands for the pure synthetic PAF102 indicating a tendency to form multimers (Figure 7B). Thus, our data suggest that PAF102 is released from the fusion protein and associates with other compounds in OB fractions or multimerizes.

To characterize the released PAF102 peptide, we tested the antifungal activity of the different OB fractions in *in vitro* fungal growth inhibitory assays (Figures 7C–E). First, we checked whether the synthetic PAF102 was active against *F. proliferatum* in the OB isolation buffer (Figure 7C). We observed total fungal growth inhibition at the concentration of 4 μM PAF102. This inhibitory concentration agrees with reported values (López-García et al., 2015). Then, we tested intact OBs carrying the Ole18-PAF102 fusion protein from two independent lines in comparison to OBs from empty vector lines, and we did not detect fungal growth inhibitory activity (Figure 7D). This is an additional evidence that the fusion protein Ole18-PAF102 has no antifungal activity, as suggested by the fungal infection assays of the seeds accumulating the fusion protein (Figure 6). Finally, OB fractions digested with TEV protease containing the released PAF102 showed clear growth inhibitory activity against *F. proliferatum*, whereas the empty vector fractions did not possess antifungal activity (Figure 7E). OB fractions from line *pOle18:Ole18-PAF102* #6 showed higher inhibitory capacity than those from line #3, in agreement with the protein accumulation levels. The inhibitory activity depicted by fractions from line #6 was similar to wild-type fractions supplemented with the synthetic PAF102. According to the activity, the amount of PAF102 in OB fractions was around 3–4 μM (30 μl from a total of 100 μl obtained from 10 seeds), which represents 13.3–17.6 μg/g of seed. These values agree with the estimation for the fusion protein Ole18-PAF102, which was 15 ± 6 μg/g of seed. Therefore, the results indicate that most of the PAF102 was released after the proteolytic digestion of the Ole18-PAF102 fusion protein as an active antifungal peptide.

## Discussion

Our study demonstrates that rice seeds can be used as biofactories of rationally designed antifungal peptides, exemplified in the PAF102. The production of this small bioactive peptide was only feasible when fused to the Ole18 protein, and its accumulation was not detected when expressed as a single peptide. Since the identification of PAF26 (López-García et al., 2002), this and other PAF-derived peptides have been recalcitrant to be produced through biotechnology (either in bacteria, yeast, or higher eukaryotes). Therefore, one major point of novelty of the current study is to manage the biotechnological production of PAF peptides through fusion to oleosin. We have defined a model for the mode of action of fungal-specific and PAF-derived peptides in three steps: interaction with fungal cells, internalization, and intracellular killing (Muñoz et al., 2013; López-García et al., 2015). This implies that the specificity of these peptides relies in the interaction with the fungal cell envelope and the subsequent internalization. Once the peptide is inside the cell, it might be active and killing other cells different than fungal cells, such as bacteria cells. We speculate that the peptide produced in any cell factory could be toxic when it accumulates intracellularly, unless fused to a carrier such as the oleosin, as demonstrated in this study. The fusion of PAF102 to the Ole18 protein targeted the peptide to the OBs, where it remains stable during long-time storage and accumulates to high amounts of up to 20 μg/g of seeds for such small peptides (3.9 kDa).

The production of PAFs as single peptides was approached using three different promoters, namely *pGluB1*, *pGluB4*, and *pGlb1*, which drive strong endosperm-specific expression. Our approach was based on targeting the peptide to PBs with the three corresponding seed storage protein signal peptides and the KDEL extension to produce the derivative PAF103. These SPs are known to guide protein sorting into PBs, including antimicrobial peptides (Müntz, 1998; Yang et al., 2003; Ibl and Stoger, 2012; Bundó et al., 2014; Montesinos et al., 2017; Takaiwa et al., 2017). The KDEL extension was introduced because it normally increases protein accumulation levels in PBs (Takagi et al., 2005b; Takaiwa et al., 2017). The two glutelin promoters, namely *pGluB1* and *pGluB4*, have been reported to direct expression to the outer endosperm and have been successfully used to express the *Cecropin A* gene, encoding a small, linear, and cationic antimicrobial peptide (Bundó et al., 2014). The globulin promoter directs the expression to the inner endosperm and worked better than *pGluB1* and *pGluB4* for the expression of the *BP178* gene, encoding also a synthetic small, linear, and cationic antimicrobial peptide (Montesinos et al., 2017). Although we showed that all the three promoters directed the expression of the gene to the rice seeds, we could not detect the product using different methods. Failed detection supports that the peptide did not accumulate in the rice seed. Given that all the rice plants expressing the *PAF* transgenes showed normal growth and development, and no altered seed filling and yield, cytotoxic effects seem not to be responsible for the lack of PAF accumulation. The most plausible reason is peptide instability in plant tissues. Further experiments are needed to understand why these peptides are not accumulated in rice seeds. Whatever was the reason, our results clearly show difficulties to produce PAFs as single peptides in rice seeds.

A better strategy for the production of this type of peptides is the fusion to the Ole18 protein. The fusion protein guided by the oleosin is embedded in OBs where peptides are immobilized and inactivated. Our assays clearly show that Ole18-PAF102 does not exhibit *in vivo* or *in vitro* activity against fungi, but as soon as it is released from the Ole18 it becomes active. The immobilization in OBs confers protection and offers stability to PAF102, allowing its accumulation. In our experiments, the amount of PAF102 reached up to 20 μg/g of seed, which taking into account the low molecular weight of the peptide (3.9 kDa), corresponds to 5.1 nmoles/g of seed. This yield is a little lower than the one obtained with cecropin A using the same production strategy (8 nmoles/g) (Montesinos et al., 2016), but still on the average of reported yields for small peptides in rice seeds (0.03–10 nmoles/g of seed) (Yasuda et al., 2005; Takagi et al., 2005a,b, 2008, 2010; Suzuki et al., 2011; Wakasa et al., 2011; Wang et al., 2013), or even proteins, such as lysozyme yielding up to 80 μg/g (5.6 nmoles/g of seed) when produced from a single expression cassette or 150 μg/g (10.5 nmoles/g of seed) from two independent expression cassettes (Hennegan et al., 2005). Most of these recombinant proteins were produced in the rice endosperm, which accounts for most of the grain volume (90%), whereas our strategy of protein accumulation in OBs is restricted to the rice embryo and the aleurone layer that represents only 10% of the rice grain volume. It would be interesting to explore the production of PAF102 in OB-rich seeds such as safflower, sesame, rapeseed, soybean, or sunflower. These oily seeds have been used to produce recombinant proteins using the oleosin fusion technology (Parmenter et al., 1995; Boothe et al., 2010; Nykiforuk et al., 2011). Reported yield ranges from 0.13% of total protein for insulin in *Arabidopsis thaliana* seeds (Nykiforuk et al., 2006), 0.27% for hirudin in *Brassica napus* seeds (Parmenter et al., 1995), to 0.55% for human growth factor protein in safflower (Boothe et al., 2010). Taking into account that rice grain is not particularly rich in OBs, and our PAF yield in rice is 0.025% of seed proteins, we predict that commercial relevant values might be reached in oily seeds. Therefore, our proof-of-concept study indicates that the technology of oleosin fusion might be the best strategy to produce PAF peptides with the projection to improve yields in oily crops.

In addition to offer stability, OB accumulation facilitates the purification of PAFs from plant material by simple flotation in dense sucrose solutions. However, the PAF102 immobilized in the OBs was not active and required to be released from the Ole18 for activity. Equally, the antimicrobial peptide CecA did not exhibit activity while immobilized on the OBs (Montesinos et al., 2016), whereas other proteins have been reported to be active while associated to OBs, such as the ß-glucuronidase (van Rooijen and Moloney, 1995), a xylanase (Liu et al., 1997; Hung et al., 2008), the D-hydantoinase (Chiang et al., 2006), or the D-psicose-3-epimerase (Tseng et al., 2014), among others. The lack of activity shown by the antimicrobial peptide PAF102 while immobilized in OBs might be related to its mode of action. Being attached to OBs might prevent the PAF102 internalization into fungal cells, a process that is required for its antifungal action (Muñoz et al., 2013; López-García et al., 2015). Appropriately, antifungal activity was recovered upon release from OBs by TEV protease digestion, exhibiting equivalent activity against *F. proliferatum* to the synthetic peptide. Therefore, our results show that biologically active PAFs can be produced *in planta*.

Although rice seeds are starchy more than oily, and they might not be the best host for production of oleosin fusion proteins, they offer unique opportunities as bioreactors since the rice gene transfer technology is well developed, cropping conditions are easy and well-established worldwide, and high grain yield can be obtained (Stoger et al., 2005; Takaiwa et al., 2017). Moreover, the accumulation in seeds provides long-term stability during storage at room temperature, up to 3 years in the case of PAF102. Seeds can be stockpiled without the need to synchronize production with product demand. Additionally, the OBs are restricted to the embryo cells and aleurone layer in the rice grain. Thus, the production of the Ole18-PAF fusion protein driven by the *Ole18* promoter was only found in these specific tissues. Along with the embryo, the seed coats, including aleurone and pericarp, are separated during rice milling to obtain the white refined grain and remain as the rice bran by-product. Consequently, downstream purification is facilitated using the PAF102-enriched rice bran as the starting plant material. The use of rice bran for the production of PAFs could add an extra value to this by-product assisting their exploitation to bring them to market.

## Ethics Statement

Mouse polyclonal antibodies were produced at the Laboratory Animal Facilities (registration number B9900083) of the Center for Research and Development (CID) from Spanish National Research Council (CSIC), in strict accordance with the bioethical principles established by the Spanish legislation following international guidelines. The protocol was approved by the Committee on Bioethics of Animal Experimentation from CID and by the Department of Agriculture, Livestock, Fisheries, Food and Environment of the Government of Catalonia (permit number DAAM:7461). All efforts were made to minimize suffering of the animals.

## Author Contributions

JM, BL-G, and MC conceived and designed the study. BL-G and MB prepared the gene constructs to be introduced in rice. MB carried out all rice transformation experiments and the molecular characterization of transgenic plants. MB, MV, and MC characterized phenotypically the generated transgenic rice plants. XS, MV, and MC characterized PAF production in rice seeds. MC coordinated the study and prepared the manuscript. All the authors read and approved the final manuscript.

### Conflict of Interest Statement

The authors declare that the research was conducted in the absence of any commercial or financial relationships that could be construed as a potential conflict of interest.
