# Supplementary material for: Rice Seeds as Biofactories of Rationally Designed and Cell-Penetrating Antifungal PAF Peptides
Source: Front Plant Sci. 2019 Jun 7;10:731. doi: 10.3389/fpls.2019.00731 (PMC6566136; doi:10.3389/fpls.2019.00731)
Supplement: Supplementary file 1 [file Presentation_1.pdf]

Supplementary Table 1. Primers used in this study

Primers used for cloning and PCR analysis. Restriction sites are underlined.

NarIPAF103_fwd	5´ATTA**GGCGCC**CACCACCACCACCACCACCGCAAGAAGT3’
PAF103NarI_rev	5’GTGCAC**GGCGCC**ATTACTAGAGTTCGT3´
promOle18_fwd	5’GG**GAATTC**GATGGTCAGCCAATACATTGATCCGTT3’
Ole18_rev	5’TCT**GTCGAC**GATGTCTTGGTGCCGGC3’
HindIII_tNos_rev	5’TG**AAGCTT**GTTTGACAGGTTATCATCGGATCTAGTAACATAG3’
PstI_tNos_fwd	5’TT**CTGCAG**CCCGGGGATCGTTCAAACATTTG3’
promGluB1_fwd	5'GG**GGTACC**TCTAGACAGATTCTTGCTACCAA3'
promGluB4_fwd	5'GG**GGTACC**TACAGGGTTCCTTGCGTGAAGAA3'
promGlb1_fwd	5’**GGTACC**TGGAGGGAGGGAGAGGGGAGAGATG3’
SacI_tNos_rev	5'CC**GAGCTC**GTTTGACAGCTTATCATCGGATCTA3‘

Primers used for RT-PCR analysis

OsEF1a_fwd	5’GTGCTCGACAAGCTCAAGGCCG3’
OsEF1a_rev	5’GTCTGATGGCCTCTTGGGCTCG3’
SPGluB1PAF_fwd	5’TGGCGAGTTCCGTTTTCTCT3’
SPGluB1PAF_rev	5’GTTCGTCCTTCCAGAACCACT3’
SPGluB4PAF_fwd	5’TGGCGACCATAGCTTTCTCTC3’
SPGlb1PAF_fwd	5’AGCAAGGTCGTCTTCTTCGC3’
SPGlb1PAF_rev	5’CCACTTCTTGCGGCGG3’

Supplementary Table 2. Estimation of transgene copy number by qPCR analysis. Values correspond to the Ct mean and standard deviation of qPCR analysis. The amplicons were the rice single copy *SPS* gene and the t-Nos region of the transgene.

		SPS		*Tnos*		
		Mean	SD	Mean	SD	Copy number
	Wt	22.06	0.11	31.75	0.24	0.69
	EV	22.34	0.20	31.33	0.28	0.71
pOle18:Ole18-PAF102: Tnos	1	25.49	0.08	25.95	0.20	0.98
	3	22.14	0.07	23.18	0.03	0.95
	6	22.20	0.09	24.11	0.07	0.92
	5	22.40	0.08	22.56	0.05	0.99
	7	21.91	0.02	19.90	0.05	1.10
pGluB4:PAF103: Tnos	1.2	22.09	0.06	20.82	0.02	1.06
	3.1	22.43	0.10	19.47	0.05	1.15
	3.4	21.98	0.10	19.72	0.02	1.11
	1.4	22.11	0.08	20.04	0.06	1.10
pGluB1:PAF103:Tnos	5	22.28	0.09	19.70	0.06	1.13
	8	25.79	0.06	22.51	0.27	1.15
	1	22.67	0.10	20.76	0.12	1.09
	3	22.52	0.05	20.51	0.16	1.10
	13	22.78	0.08	21.38	0.19	1.07
pGlb1:PAF103:Tnos	1	31.67	0.01	21.78	0.22	1.45
	6	26.81	1.25	22.80	1.19	1.18
	7.1	25.46	0.01	22.45	0.24	1.13
	7.2	25.60	0.66	22.01	0.00	1.16

frontiers

>>PAF103

ggatccatggcc**CACCACCACCACCACCACCACCGCAAGAAGTGGTTCTGGGCCG**
GCCCGGCCCGCCGCAAGAAGTGGTTCTGGGCCGGCCCGGCCTGGCGCAAGAAGTG
GTTCTGGAAGGACGAACTCTAGTAAt**ggatcc**

>>PAF102

gtcgacaCCGACCACCGAGAACCTCTACTTCCAGGGCCACCGCAAGAAGTGGTTC
TGGGCCGGCCCGGCCCGCCGCAAGAAGTGGTTCTGGGCCGGCCCGGCCTGGCGCA
AGAAGTGGTTCTGGTAGTAAt**ctgcag**

Supplementary Figure 1. DNA sequence of the synthetic *PAF103* and *PAF102* genes. Underlined sequences are the His-tag and KDEL-extension encoding sequences. Blue color sequences are the protease recognition site (PRS). Green color sequences are the restriction enzyme recognition sites used for cloning purposes.

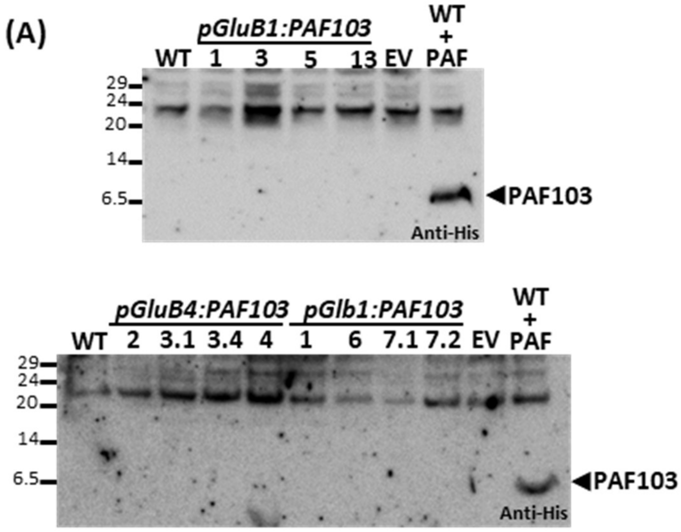

Supplementary Figure 2. PAF103 does not accumulate in rice seeds. Immunoblot analysis of PAF103 using anti-His monoclonal antibodies, in PB enriched fractions purified from wild-type (WT), or from transgenic homozygous mature seeds carrying the empty vector (EV), or the indicated transgenes. As a positive control, synthetic PAF103 peptide was added to WT extracts and run in parallel (WT+PAF).

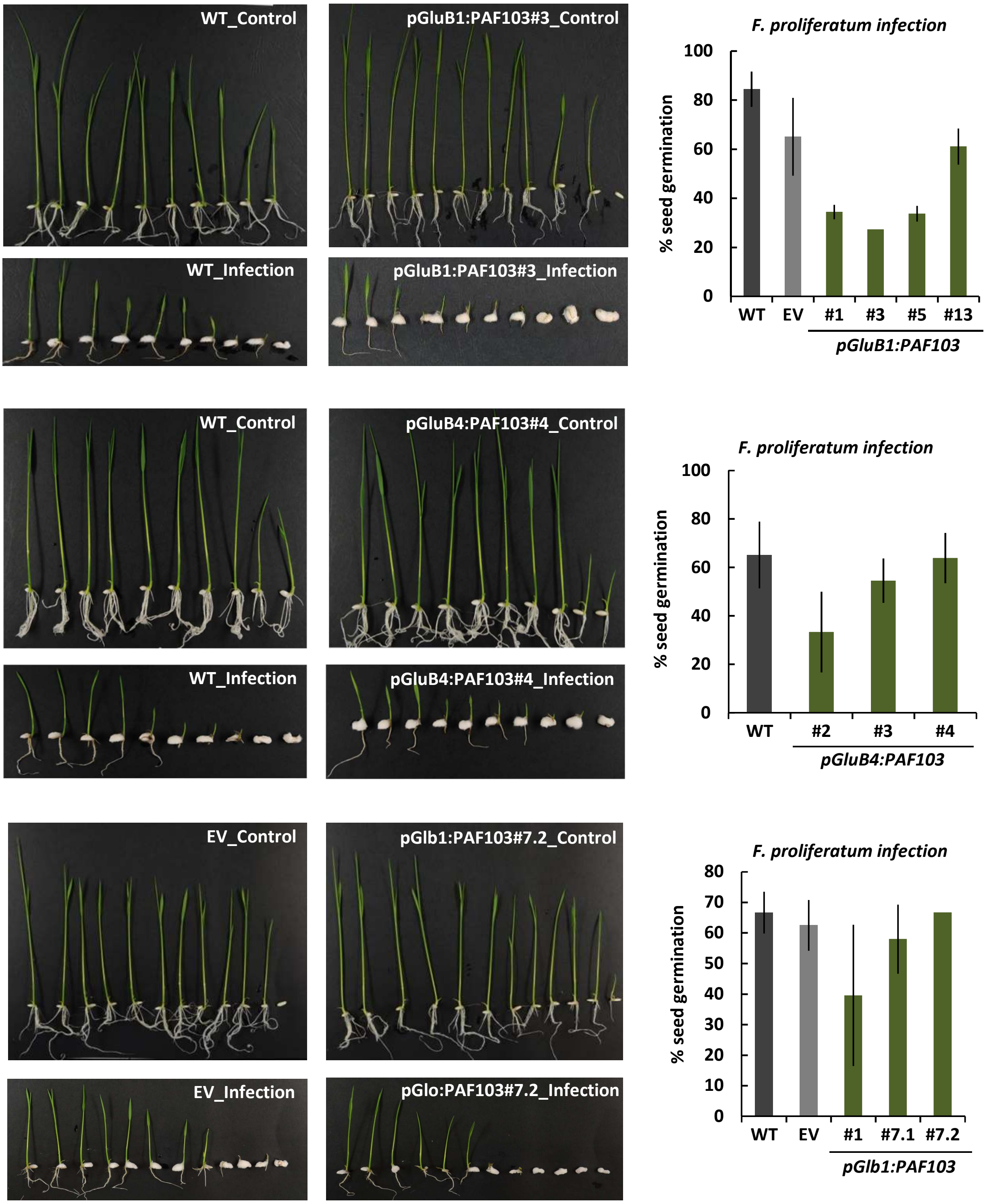

Supplementary Figure 3. Fungal infection assays of *PAF103* transgenic seeds with the phytopathogen *F. proliferatum.* Phenotypical appearance of wild-type (WT) and transgenic seedlings carrying the empty vector (EV) or the indicated transgenes, at 7 days after germination under control conditions or inoculated with *F. proliferatum* spore suspension (10^3 spores/ml). Pictures are representative of at least 3 independent lines per construct, and at least 3 independent assays. The graphs show the mean and standard deviation values of germination rate of 3 independent assays.
